# Trends in Intracranial Stenting Among Medicare Beneficiaries in the United States, 2006–2010

**DOI:** 10.1161/JAHA.113.000084

**Published:** 2013-04-24

**Authors:** Aakriti Gupta, Mayur M. Desai, Nancy Kim, Ketan R. Bulsara, Yun Wang, Harlan M. Krumholz

**Affiliations:** 1 Center for Outcomes Research and Evaluation, Yale‐New Haven Hospital, New Haven, CT (A.G., M.M.D., N.K., Y.W., H.M.K.); 2 Section of Cardiovascular Medicine, Department of Internal Medicine, Yale University School of Medicine, New Haven, CT (A.G., H.M.K.); 3 Section of Chronic Disease Epidemiology, Yale School of Public Health, New Haven, CT (M.M.D.); 4 Robert Wood Johnson Clinical Scholars Program, Department of Internal Medicine, Yale University School of Medicine, New Haven, CT (M.M.D., H.M.K.); 5 Section of General Internal Medicine, Department of Internal Medicine, Yale University School of Medicine, New Haven, CT (N.K.); 6 Department of Neurosurgery, Yale University School of Medicine, New Haven, CT (K.R.B.); 7 Department of Biostatistics, Harvard School of Public Health, Boston, MA (Y.W.); 8 Section of Health Policy and Administration, Yale School of Public Health, New Haven, CT (H.M.K.)

**Keywords:** device exemption, elderly, intracranial stenting, technology adoption, trends

## Abstract

**Background:**

It is uncertain how intracranial stenting (ICS) has been adopted nationally during a period characterized by a restrictive payment policy by the Centers for Medicare & Medicaid Services, humanitarian device exemption approval by the Food and Drug Administration, and insufficient evidence of effectiveness. We sought to determine the trends in rates of ICS use and associated outcomes in the United States.

**Methods and Results:**

From 65 211 328 Medicare Fee‐for‐Service beneficiaries hospitalized between 2006 and 2010 in acute care hospitals in the United States, we included patients with ICD‐9‐CM procedure codes for intracranial angioplasty and stenting, excluding those with a principal discharge diagnosis code of cerebral aneurysm or subarachnoid hemorrhage. We report operative rates per 1 000 000 person‐years and outcomes including 30‐day and 1‐year mortality rates. There were 838 ICS procedures performed among Fee‐for‐Service beneficiaries. The overall hospitalization rate for ICS increased significantly from ≈1 per 1 000 000 person‐years (n=35 procedures) in 2006 to 9 per 1 000 000 person‐years (n=258 procedures) in 2010 (*P*=0.0090 for trend). Procedure rates were higher in men than in women, and were highest among patients aged 75 to 84 years and lowest among those ≥85 years. The 30‐day mortality rate increased from 2.9% (95% CI, 0.1 to 15.3) to 12.9% (95% CI, 9.0 to 17.6), *P*=0.1294 for trend, and the 1‐year mortality rate increased from 14.7% (95% CI, 5.0 to 31.1) to 19.5% (95% CI, 14.9 to 24.9), *P*=0.0101; however, the annual changes were not significant after adjustment.

**Conclusions:**

ICS utilization in the United States has modestly increased during a period of inadequate supportive evidence. Humanitarian device exemption and a restrictive payment policy appear to have caused slow adoption of the technology.

## Introduction

Intracranial atherosclerosis causes 8% to 10% of all ischemic strokes in the United States^1–2^ and is associated with a high rate of recurrent stroke. Given the persistent risk of recurrence despite optimal medical management strategies,^3–4^ there is strong interest in alternative treatment options such as intracranial stenting (ICS). The Wingspan stent, designed specifically for use in intracranial atherosclerotic disease, was granted humanitarian device exemption (HDE) approval by the United States Food and Drug Administration (FDA) in August 2005.^5^ HDE waives the requirement that a manufacturer submit a premarket approval application to demonstrate a product's effectiveness through scientifically valid clinical investigations. The device was indicated for “improving cerebral artery lumen diameter in patients with ≥50% stenosis, refractory to medical therapy that is accessible to the system.”^5^

The lack of evidence about the procedure engendered some controversy. A joint position statement^6^ by major radiology and neuroradiology societies in October 2005, in alignment with the FDA indication, concluded that balloon angioplasty with or without stenting should be considered in a subgroup of patients who had failed medical therapy. However, the studies that were available included individual reports and case series that were not designed to demonstrate the superiority of stenting over conventional medical management.^7–10^ A Cochrane review in 2006^11^ stated that the evidence precluded any conclusions about the effectiveness of the procedure. The same year, the Centers for Medicare & Medicaid Services (CMS) provided coverage for this procedure only in the context of a randomized trial.^12^ In 2011, published results of the Stenting and Aggressive Medical Management for Preventing Recurrent stroke in Intracranial Stenosis (SAMMPRIS) trial demonstrated that intracranial angioplasty and stenting more than doubled the risk of stroke or death within 30 days compared with medical management alone in patients who had a recent stroke or transient ischemic attack.^13^ This risk remained elevated even at 1 year, with a mean follow‐up period of 11.9 months.

To determine the adoption pattern of this new technology, we analyzed a 100% sample of Medicare Fee‐for‐Service beneficiaries to determine national trends in ICS utilization rates and outcomes among patients with intracranial atherosclerotic disease from 2006 to 2010, the period during which there was no randomized trial evidence. We also sought to study a subgroup of patients who met the eligibility criteria for enrollment in SAMMPRIS to estimate the pattern of use in this cohort since the approval of the technology.

## Methods

### Data Sources and Coding

We used data from Medicare inpatient standard analytical files from CMS to identify all Fee‐for‐Service beneficiaries who were hospitalized for intracranial angioplasty and stenting between January 1, 2006, and December 31, 2010. We defined ICS using International Classification of Diseases, Ninth Revision, Clinical Modification procedure codes for both intracranial angioplasty (00.62) and ICS (00.65). We excluded beneficiaries younger than 65 years and those with a principal discharge diagnosis code of subarachnoid hemorrhage (430) or cerebral aneurysm (437.3).

For secondary analyses, we further defined a subgroup of patients who were most comparable to those meeting eligibility criteria for enrollment in SAMMPRIS. From the primary sample, we identified hospitalizations that had principal discharge diagnosis codes for stroke (434.×1) or transient ischemic attack (435.0, 435.1, 435.3, 435.8, and 435.9) within 30 days of or during index admission. We ascertained dates of death through the corresponding vital status information in the denominator files.

### Patient Characteristics and Comorbidities

We examined demographic characteristics of patients who underwent ICS, including age, sex, and race. We determined race using the Medicare denominator file, which uses patient‐reported data from the Social Security Administration.^14^ We identified clinical comorbidities using secondary diagnosis codes that did not represent potential complications (Appendix S1) from the initial ICS hospitalization as well as principal and secondary diagnosis codes of all hospitalizations for any cause in the 12 months before the initial ICS hospitalization.

### ICS Hospitalization Rates and Outcomes

For each year, we calculated the ICS hospitalization rate by dividing the total number of ICS hospitalizations by the total accumulated person‐years. Because in a given year some beneficiaries may be enrolled in Medicare Fee‐for‐Service for <12 months, we calculated the total number of beneficiary‐months at risk and then converted to person‐years for the denominator.

To calculate in‐hospital, 30‐day, and 1‐year mortality, we identified all hospitalizations that occurred for ICS in a given year. If a patient had multiple hospitalizations for ICS in a given year, we selected 1 at random, consistent with methods used to calculate CMS publicly reported mortality measures. The procedure date of that hospitalization represented the “time zero” for the mortality analysis. As a result, the 30‐day and 1‐year mortality rates represented the likelihood of death within 30 days and 1 year of the procedure among patients hospitalized for ICS in a given calendar year. To generate 30‐day readmission rates, we restricted the sample to patients who were discharged alive and not transferred to another acute care hospital. The date of index discharge represented “time zero” for readmission.

### Statistical Analysis

We expressed the ICS hospitalization rate as per 1 000 000 person‐years, mortality and readmission rates as percentages, and length of stay as mean (standard deviation [SD]) days. We used the Mantel–Haenszel chi‐square test to analyze whether changes over time in the outcomes were statistically significant. To assess the annual change in ICS hospitalization rates, we fitted a linear mixed‐effects model with a Poisson link function and state‐specific random intercepts, adjusted for demographics. For this analysis, we included a continuous time variable, ranging from 0 to 4, corresponding to years 2006–2010, to estimate the risk‐adjusted incidence rate ratio (IRR) that represents the annual change in ICS hospitalizations during that period. To obtain the annual change in mortality rates adjusted for patient demographics and comorbidities, we fitted a linear mixed‐effects model with a logit link function and state‐specific random intercepts. We used the time variable, described previously, to calculate the risk‐adjusted odds ratio (OR) that represents the annual change in ICS mortalities from 2006 to 2010.

For 30‐day all‐cause readmission rates, we conducted survival analysis to calculate the proportion of patients who were readmitted to any hospital within 30 days of discharge for the index ICS procedure, censoring those who died before readmission. We constructed a Cox proportional model with state‐specific random intercepts and the time variable to evaluate the annual changes in 30‐day all‐cause readmission rates over time adjusted for patient demographics and comorbidities.

We conducted the analyses with SAS version 9.3 64‐bit (SAS Institute Inc, Cary, NC). The significance level for all analyses was *P*<0.05 using 2‐sided tests. The Institutional Review Board at Yale University approved the study.

## Results

### ICS Hospitalizations and Patient Characteristics

**Figure 1.**
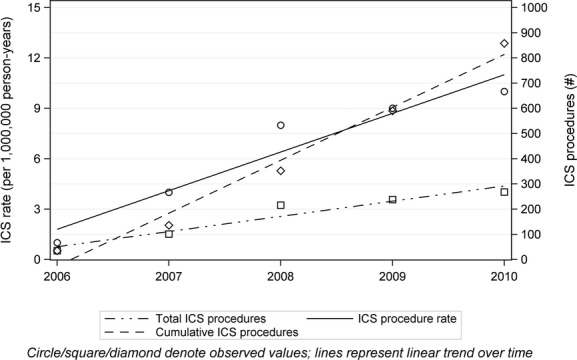
Left *y* axis corresponds to the intracranial stenting (ICS) procedure rate. Right *y* axis corresponds to total ICS procedures and cumulative ICS procedures.

The final sample included 146 459 811 observations in the denominator files from 2006 to 2010, representing 65 211 328 individual Medicare beneficiaries who contributed a total of 139 067 831 person‐years of observation. There were 838 ICS procedures contributed by 826 individual Medicare Fee‐for‐Service beneficiaries performed during the study period (Table 1). The overall hospitalization rate for ICS increased significantly, from ≈1 per 1 000 000 person‐years (n=35 procedures) in 2006 to 9 per 1 000 000 person‐years (n=258 procedures) in 2010 (*P*=0.0090 for trend; Figure 1). After controlling for age, sex, and race, the risk‐adjusted IRR that represents the relative annual change in the ICS hospitalization rate was 1.72 (95% confidence interval [CI], 1.69 to 1.76). Increases in ICS rates were observed across all age, sex, and race subgroups (Table 1). The hospitalization rate for ICS was higher in men than in women throughout the study period, with 2010 rates of 12 per 1 000 000 and 7 per 1 000 000, respectively. In 2010, the ICS rate was observed to be highest in patients aged 75 to 84 years (11 per 1 000 000), followed by those aged 65 to 74 years (10 per 1 000 000) and ≥85 years (5 per 1 000 000). The ICS hospitalization rate was comparable among blacks and whites across the study period, with a rate of 9 per 1 000 000 for both in 2010.

**Table 1.** ICS Hospitalization Rates of Medicare Fee‐for‐Service Beneficiaries, 2006–2010

	2006	2007	2008	2009	2010
Person‐years	28 452 501	27 899 732	27 675 586	27 343 436	27 696 576
ICS Hospitalizations (n)	35	101	216	228	258
Rate of ICS (per 1 000 000 person‐years)	1	4	8	8	9
By age* (y)					
65 to 74	1	3	7	8	10
75 to 84	2	4	9	10	11
≥85	0	2	6	5	5
By sex*					
Male	2	5	10	10	12
Female	1	3	7	7	7
By race*					
White	1	4	8	8	9
Black	1	4	11	10	9
Other*	1	2	6	8	12

ICS indicates intracranial stenting.

* Rates within subgroups reported per 1 000 000 person‐years.

* Includes Asian, Hispanic, North American Native, and other not specified.

Over time, the mean age, sex, and race of patients did not vary substantially. Between 2006 and 2010, there was a decrease in prevalence for some comorbidities, most notably hypertension (85.3% to 75.0%; *P*=0.0776 for trend), diabetes mellitus (41.2% to 27.7%; *P*=0.1953 for trend), and atherosclerotic disease (52.9% to 31.3%; *P*=0.0222, for trend).

### Mortality Outcomes

**Figure 2.**
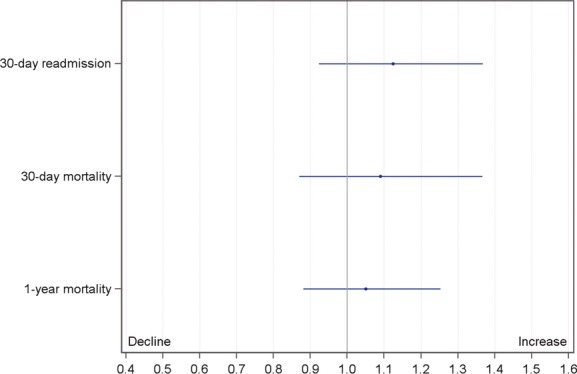
Adjusted odds ratios for annual change in outcomes in patients receiving intracranial stenting among Medicare Fee‐for‐Service beneficiaries during 2006–2010.

The overall in‐hospital mortality rates were 2.9% (95% CI, 0.1% to 15.3%) and 9.0% (95% CI, 5.8% to 13.2%) in 2006 and 2010, respectively (Table 2). The overall 30‐day mortality rate for ICS patients was 2.9% (95% CI, 0.1% to 15.3%) in 2006 and 12.9% (95% CI, 9.0% to 17.6%) in 2010 (Table 2). The annual change in 30‐day mortality rate was not significant after adjustment for patient demographics and comorbidities (OR, 1.09; 95% CI, 0.87 to 1.36; Figure 2).

**Table 2.** Outcomes Among Patients Undergoing ICS in Medicare Fee‐for‐Service, 2006–2010

	2006	2007	2008	2009	2010
LOS in days, mean (SD)	3.6 (4.9)	4.6 (4.7)	4.8 (7.7)	3.8 (4.7)	5.7 (8.3)
30‐day readmission, %	6.1 (0.7 to 20.2)	12.5 (6.4 to 21.3)	10.8 (6.8 to 16.0)	7.2 (4.1 to 11.6)	14.7 (10.4 to 19.9)
In‐hospital mortality, %	2.9 (0.1 to 15.3)	8.3 (3.7 to 15.8)	7.5 (4.4 to 12.0)	7.9 (4.8 to 12.2)	9.0 (5.8 to 13.2)
30‐day mortality, %	2.9 (0.1 to 15.3)	11.5 (5.9 to 19.6)	9.9 (6.2 to 14.7)	11.4 (7.6 to 16.3)	12.9 (9.0 to 17.6)
1‐year mortality, %	14.7 (5.0 to 31.1)	14.6 (8.2 to 23.3)	17.5 (12.6 to 23.2)	18.4 (13.6 to 24.1)	19.5 (14.9 to 24.9)

ICS indicates intracranial stenting; LOS, length of stay; SD, standard deviation.

The 1‐year mortality rate for ICS increased from 14.7% (95% CI, 5.0% to 31.1%) in 2006 to 19.5% (95% CI, 14.9% to 24.9%) in 2010, but this increase was not significant after adjustment for patient demographics and comorbidities (OR, 1.05; 95% CI, 0.88 to 1.25; Figure 2). The rates were highest for those aged 85 years and older (35.7% in 2010), followed by the 75‐ to 84‐ and 65‐ to 74‐year age groups (2010, 20.0% and 15.8%, respectively). In 2010, women had higher 1‐year mortality than men (23.3% versus 16.4%). Nonetheless, 1‐year mortality rates increased across the subgroups of age, sex, and race throughout the study period.

### Readmission and Length of Stay

The 30‐day readmission rate was 6.1% (95% CI, 0.7% to 20.2%) in 2006, compared with 14.7% (95% CI, 10.4% to 19.9%) in 2010 (Table 2). The annual increase in the 30‐day readmission rate was not significant after adjustment for patient demographics and comorbidities (OR, 1.12; 95% CI, 0.92 to 1.36; Figure 2). Over time, mean (SD) hospital length of stay increased from 3.6 (4.9) to 5.7 (8.3) days (*P*=0.1507 for trend).

### Subgroup Analyses

We identified 310 patients during 2006–2010 who met eligibility criteria for enrollment in SAMMPRIS. The number of ICS procedures performed in this subgroup increased from 6 in 2006 to 107 in 2010. The overall 30‐day readmission rate in this subgroup was 10.3%, 30‐day mortality rate was 18.6%, and 1‐year mortality rate was 26.5%.

Among the 310 patients who met eligibility criteria for SAMMPRIS, 202 were admitted with stroke and 108 with a transient ischemic attack within 30 days preceding admission. The overall 30‐day readmission rates were 9.9% in patients with stroke and 11.1% in patients with transient ischemic attack during 2006–2010. The 30‐day mortality rates were 17.3% and 21.3% and the 1‐year mortality rates were 25.7% and 27.8% among patients with stroke and transient ischemic attack, respectively.

## Discussion

During a period of uncertainty about the efficacy and safety of intracranial angioplasty and stenting, CMS imposed a highly restrictive payment policy that limited reimbursement to patients who were enrolled in a randomized trial.^12^ We found limited adoption of the technology from 2006 to 2010, although during that time there were 826 Medicare Fee‐for‐Service beneficiaries who received ICS. This group may have included some patients from SAMMPRIS, but the number is likely to have been small given that there were only 224 patients, with an average age of 61.0 years, recruited into the ICS arm of SAMMPRIS.^13^

Under an HDE application for patients who are refractory to medical therapy, the Wingspan Stent System was approved by the FDA in 2005 on the basis of a single, uncontrolled, 45‐subject trial that was not designed to demonstrate whether utilization of the device was safer or more effective than medical therapy alone.^15–16^ Most of the studies conducted before and after the Wingspan device was approved either had no control group that received medical therapy alone^15,17–22^ or involved comparison with a historical^9,23^ or a nonrandomized^24^ control group. Thus, valid conclusions could not be drawn regarding the relative safety or efficacy of stenting over standard medical therapy alone. During a time of uncertainty surrounding rational utilization of ICS in patients with intracranial atherosclerotic disease and lack of an evidence base demonstrating its efficacy over medical therapy, our study demonstrates a small absolute increase in adoption of the procedure over recent years.

SAMMPRIS, the only published clinical trial of this technology, was published 6 years after the stent was approved.^13^ Recruitment was prematurely discontinued after the finding that the 30‐day rate of stroke or death in patients randomized to the ICS arm was nearly 2.5 times that of patients who received aggressive medical management alone. Notably, although the inclusion of patients in SAMMPRIS did not require refractoriness to medical therapy, about two thirds of the patients were on antithrombotic therapy at the time of randomization. In addition, many patients who would not have qualified for the SAMMPRIS trial also received this procedure. Because of lack of clinical data, it is not possible to ascertain whether they were symptomatic. However, criteria for recruitment in SAMMPRIS were not in alignment with the FDA indication for ICS, and that may partly account for this observation. As per the FDA indication for ICS, refractory medical therapy was not clearly defined, whereas SAMMPRIS recruited patients within 30 days of stroke or transient ischemic attack.

CMS may be credited for the regulated adoption of this technology that lacked sufficient evidence for its use. After its decision in 2006 to offer coverage for ICS only when it is furnished in accordance with FDA‐approved protocols governing investigation device‐exemption clinical trials, CMS maintained this judgment despite a petition for broader coverage filed in 2008 by Boston Scientific.^25^ In comparison, endovascular devices for mechanical embolectomy in acute stroke patients were approved through the FDA's 510(k) pathway and are fully reimbursed by CMS, leading to a much more rapid rate of increase in their use in recent years.^26^ These instances demonstrate how regulatory bodies like CMS and the FDA can act as gatekeepers for the diffusion of a technology into clinical practice that may have considerable implications on the quality and cost of health care.^27^

Our database did not allow comparison with patients who received medical management alone, restricting any conclusion regarding safety or efficacy of this procedure. Notably, the 30‐day mortality rate that we observed among recipients of ICS in 2010 was 12.9%, much higher than the rates reported by previous observational prospective and registry studies (0% to 6%).^28^ Our study included only patients who were aged ≥65 years, unlike previous work that included younger patients. This could partly account for the discordance in outcomes. It is also possible that the outcomes of intracranial stenting are worse in practice than previously documented.

In light of results from SAMMPRIS, Public Citizen, a consumer watchdog group, petitioned the FDA in December 2011 to withdraw approval of the Wingspan Stent System.^29–30^ In response, representatives of Boston Scientific argued that SAMMPRIS was not designed to evaluate the safety of the stent in patients who are refractory to medical therapy, and thus this should not affect FDA approval for HDE‐indicated patients. Most recently, in August 2012, the FDA denied the petition, stating that it has taken regulatory actions to better define the indications and intended population for the use of the Wingspan stent, now marketed by Stryker Corp.^30^

Our study has several limitations. The analyses were limited to Medicare Fee‐for‐Service beneficiaries, which restricted the generalization of our results to patients younger than 65 years. However, ≈70% of ischemic strokes occur in patients who are older than 65 years,^31^ which supports the relevance of our focus on this subgroup. In addition, we cannot comment on trends in patients enrolled in Medicare managed care programs. As more patients have migrated into Medicare managed care programs over time,^32^ related changes in the Fee‐for‐Service population may have affected the observed trends. In addition, we could not distinguish the procedure rates and outcomes of patients who received ICS urgently from those of patients who underwent this procedure in an elective setting. Such information cannot be reliably obtained from the Medicare database. Also, we may have captured patients who received ICS for other indications. However, we excluded patients who had primary diagnoses of cerebral aneurysm and subarachnoid hemorrhage, the other 2 common indications of ICS. It is also possible that miscoding caused some basilar and vertebral artery procedures to be missed. However, coding guidelines issued by CMS require all intracranial procedures to be assigned codes for intracranial angioplasty and stenting. In addition, our study period ranged from 2006 to 2010, following the introduction of the codes for intracranial stenting and coding guidelines. Finally, we relied on administrative claims data to obtain comorbidities.

## Summary

The rate of ICS utilization in the United States modestly increased during 2006–2010, but over this period <1000 patients older than 65 years of age were treated. The approach of HDE and a restrictive payment policy appear to have caused slow adoption of the technology in the treatment of Medicare beneficiaries. Unfortunately, 8 years after approval, uncertainty remains regarding the safety and effectiveness of the device in patients who are refractory to medical therapy, even as the harm demonstrated by SAMMPRIS has justified the cautious approach taken by regulatory agencies.

## References

[1] Wong LK Global burden of intracranial atherosclerosis. Int J Stroke. 2006; 1:158-159 18706036 10.1111/j.1747-4949.2006.00045.x

[2] Gorelick PB Wong KS Bae HJ Pandey DK Large artery intracranial occlusive disease: a large worldwide burden but a relatively neglected frontier. Stroke. 2008; 39:2396-2399 18535283 10.1161/STROKEAHA.107.505776

[3] Chimowitz MI Lynn MJ Howlett‐Smith H Stern BJ Hertzberg VS Frankel MR Levine SR Chaturvedi S Kasner SE Benesch CG Sila CA Jovin TG Romano JG Comparison of warfarin and aspirin for symptomatic intracranial arterial stenosis. N Engl J Med. 2005; 352:1305-1316 15800226 10.1056/NEJMoa043033

[4] Wong KS Li H Long‐term mortality and recurrent stroke risk among Chinese stroke patients with predominant intracranial atherosclerosis. Stroke. 2003; 34:2361-2366 12947158 10.1161/01.STR.0000089017.90037.7A

[5] Approval order of Wingspan stent system. http://www.fda.gov/ohrms/dockets/dockets/05m0308/05m-0308-aav0001-approval-order-vol1.pdf.

[6] Higashida RT Meyers PM Connors JJ III Sacks D Strother CM Barr JD Wojak JC Duckwiler GR Intracranial angioplasty & stenting for cerebral atherosclerosis: a position statement of the American Society of Interventional and Therapeutic Neuroradiology, Society of Interventional Radiology, and the American Society of Neuroradiology. AJNR Am J Neuroradiol. 2005; 26:2323-2327 16221663 PMC7976119

[7] The SSYLVIA Study Investigators Stenting of symptomatic atherosclerotic lesions in the vertebral or intracranial arteries (SSYLVIA): study results. Stroke. 2004; 35:1388-1392 15105508 10.1161/01.STR.0000128708.86762.d6

[8] Gomez CR Misra VK Liu MW Wadlington VR Terry JB Tulyapronchote R Campbell MS Elective stenting of symptomatic basilar artery stenosis. Stroke. 2000; 31:95-99 10625722 10.1161/01.str.31.1.95

[9] Zaidat OO Klucznik R Alexander MJ Chaloupka J Lutsep H Barnwell S Mawad M Lane B Lynn MJ Chimowitz M The NIH registry on use of the Wingspan stent for symptomatic 70–99% intracranial arterial stenosis. Neurology. 2008; 70:1518-1524 18235078 10.1212/01.wnl.0000306308.08229.a3 PMC3506389

[10] Terada T Higashida RT Halbach VV Dowd CF Nakai E Yokote H Itakura T Hieshima GB Transluminal angioplasty for arteriosclerotic disease of the distal vertebral and basilar arteries. J Neurol Neurosurg Psychiatry. 1996; 60:377-381 8774399 10.1136/jnnp.60.4.377 PMC1073887

[11] Cruz‐Flores S Diamond AL Angioplasty for intracranial artery stenosis. Cochrane Database Syst Rev. 2006; 3:CD004133 16856032 10.1002/14651858.CD004133.pub2 PMC8764997

[12] CMS national coverage policy. http://wpsmedicare.com/j5macparta/policy/active/national/_files/cv039_cbg.pdf.

[13] Chimowitz MI Lynn MJ Derdeyn CP Turan TN Fiorella D Lane BF Janis LS Lutsep HL Barnwell SL Waters MF Hoh BL Hourihane JM Levy EI Alexandrov AV Harrigan MR Chiu D Klucznik RP Clark JM McDougall CG Johnson MD Pride GL Jr Torbey MT Zaidat OO Rumboldt Z Cloft HJ Stenting versus aggressive medical therapy for intracranial arterial stenosis. N Engl J Med. 2011; 365:993-1003 21899409 10.1056/NEJMoa1105335 PMC3552515

[14] Eicheldinger C Bonito A More accurate racial and ethnic codes for Medicare administrative data. Health Care Financ Rev. 2008; 29:27-42 18567241 PMC4195038

[15] Bose A Hartmann M Henkes H Liu HM Teng MM Szikora I Berlis A Reul J Yu SC Forsting M Lui M Lim W Sit SP A novel, self‐expanding, nitinol stent in medically refractory intracranial atherosclerotic stenoses: the Wingspan study. Stroke. 2007; 38:1531-1537 17395864 10.1161/STROKEAHA.106.477711

[16] Summary of safety and probable benefit of Wingspan stent system and Gateway PTA balloon catheter. http://www.accessdata.fda.gov/cdrh_docs/pdf5/h050001b.pdf.

[17] Henkes H Miloslavski E Lowens S Reinartz J Liebig T Kuhne D Treatment of intracranial atherosclerotic stenoses with balloon dilatation and self‐expanding stent deployment (Wingspan). Neuroradiology. 2005; 47:222-228 15912418 10.1007/s00234-005-1351-2

[18] Fiorella D Levy EI Turk AS Albuquerque FC Niemann DB Aagaard‐Kienitz B Hanel RA Woo H Rasmussen PA Hopkins LN Masaryk TJ McDougall CG US multicenter experience with the Wingspan stent system for the treatment of intracranial atheromatous disease: periprocedural results. Stroke. 2007; 38:881-887 17290030 10.1161/01.STR.0000257963.65728.e8

[19] Levy EI Turk AS Albuquerque FC Niemann DB Aagaard‐Kienitz B Pride L Purdy P Welch B Woo H Rasmussen PA Hopkins LN Masaryk TJ McDougall CG Fiorella DJ Wingspan in‐stent restenosis and thrombosis: incidence, clinical presentation, and management. Neurosurgery. 2007; 61:644-650 17881980 10.1227/01.NEU.0000290914.24976.83

[20] Fiorella DJ Turk AS Levy EI Pride GL Jr Woo HH Albuquerque FC Welch BG Niemann DB Aagaard‐Kienitz B Rasmussen PA Hopkins LN Masaryk TJ McDougall CG U.S. Wingspan Registry: 12‐month follow‐up results. Stroke. 2011; 42:1976-1981 21636812 10.1161/STROKEAHA.111.613877

[21] Yu J Wang L Deng JP Gao L Zhang T Zhao ZW Gao GD Treatment of symptomatic intracranial atherosclerotic stenosis with a normal‐sized Gateway balloon and Wingspan stent. J Int Med Res. 2010; 38:1968-1974 21227000 10.1177/147323001003800610

[22] Yu SC Leung TW Lee KT Hui JW Wong LK Angioplasty and stenting of atherosclerotic middle cerebral arteries with Wingspan: evaluation of clinical outcome, restenosis, and procedure outcome. AJNR Am J Neuroradiol. 2011; 32:753-758 21436335 10.3174/ajnr.A2363 PMC7965883

[23] Jiang WJ Yu W Du B Gao F Cui LY Outcome of patients with ≥70% symptomatic intracranial stenosis after Wingspan stenting. Stroke. 2011; 42:1971-1975 21636814 10.1161/STROKEAHA.110.595926

[24] Samaniego EA Hetzel S Thirunarayanan S Aagaard‐Kienitz B Turk AS Levine R Outcome of symptomatic intracranial atherosclerotic disease. Stroke. 2009; 40:2983-2987 19556534 10.1161/STROKEAHA.109.549972 PMC2770373

[25] Decision memo for intracranial stenting and angioplasty. http://www.cms.gov/medicare-coverage-database/details/nca-decision-memo.Aspx?Ncaid=214&ver=14&ncaname=intracranial+stenting+and+angioplasty+%285th+recon%29&bc=beaaaaaaeaaa&&fromdb=true.

[26] Khatri P Adeoye O Kleindorfer DO US rates of mechanical embolectomy for acute ischemic stroke treatment are increasing. Stroke. 2010; 41:e361

[27] Broderick JP The challenges of intracranial revascularization for stroke prevention. N Engl J Med. 2011; 365:1054-1055 21899410 10.1056/NEJMe1108394

[28] Siddiq F Memon MZ Vazquez G Safdar A Qureshi AI Comparison between primary angioplasty and stent placement for symptomatic intracranial atherosclerotic disease: meta‐analysis of case series. Neurosurgery. 2009; 65:1024-1033 19934961 10.1227/01.NEU.0000360138.54474.52

[29] Petition to food and drug administration to withdraw approval of Wingspan stent system. http://www.citizen.org/documents/petition-to-fda-to-withdraw-approval-of-wingspan-stent-system-122111.pdf.

[30] Citizen petition‐docket number FDA‐2011‐P‐0923. http://www.fda.gov/aboutfda/centersoffices/officeofmedicalproductsandtobacco/cdrh/cdrhfoiaelectronicreadingroom/ucm150022.htm.

[31] Lee LK Bateman BT Wang S Schumacher HC Pile‐Spellman J Saposnik G Trends in the hospitalization of ischemic stroke in the United States, 1998–2007. Int J Stroke. 2012; 7:195-201 22151527 10.1111/j.1747-4949.2011.00700.x

[32] Medicare advantage 2010 data spotlight: plan enrollment patterns and trends. http://www.kff.org/medicare/upload/8080.pdf.

